# Identification of Groundwater Pollution Characteristics and Health Risk Assessment of a Landfill in a Low Permeability Area

**DOI:** 10.3390/ijerph18147690

**Published:** 2021-07-20

**Authors:** Fei Wang, Kai Song, Xuelian He, Yue Peng, Dan Liu, Jian Liu

**Affiliations:** Faculty of Geosciences and Environmental Engineering, Southwest Jiaotong University, Chengdu 610031, China; wangfeikylw@163.com (F.W.); hxl960224@163.com (X.H.); p2565618550@163.com (Y.P.); liudan-swju@163.com (D.L.); liukai-102@163.com (J.L.)

**Keywords:** landfill, rapid identification of groundwater pollution, risk assessment, spatial distribution

## Abstract

The shallow weathering fissure groundwater in the red-bed area of Southwest China is usually the only drinking water source for most rural residents. In this study, a typical landfill with surrounding residents drinking unpurified groundwater in red-bed area was selected and water quality detection, groundwater numerical simulation and human health risk assessment were used to identify and assess groundwater pollution in the region. The chemical type evolved from HCO_3_-SO_4_-Ca-Mg and HCO_3_-SO_4_-Ca to Na-Ca-Cl-HCO_3_ contaminated by the landfill. Na^+^ and Cl^−^ were selected as factors for rapid identification of groundwater pollution. Subsequent analyses using these factors showed that the leachate pollution plume boundary was 190 m downstream of the landfill. Analysis of the redox conditions revealed that the area from the landfill to 5 m downstream was the reduction zone, while the area beyond 5 m was the oxidation zone. The migration and attenuation patterns of inorganic salts (such as SO_4_^2−^) and heavy metals (such as Fe and Mn) in the oxidation and reduction zones differed obviously. Meanwhile, the organic pollutants in the leachate were reduced and decomposed into organic acids, which caused the groundwater 80 m downstream of the landfill to become weakly acidic (pH ranged from 6.51 to 6.83), and promoted re-entry of adsorbed heavy metals (such as Pb) into the groundwater. The groundwater risk assessment based on human health revealed that lead, manganese, chlorobenzene, dichloroethane and chloroform constituted a major health threat to the residents. The rank of non-carcinogenic risk was lead >manganese, and the maximum area of non-carcinogenic risk was 15,485 m^2^. The total carcinogenic risk caused by organic pollutants was 7.9 × 10^−6^, and the area of the carcinogenic risk zone was 11,414 m^2^. Overall, the results of this study provide a scientific basis for management of drinking water and groundwater remediation in the red-bed area with low permeability.

## 1. Introduction

At present, landfill is the final destination of products after various methods of municipal solid waste treatment in the world [1]. There are more than 250,000 landfills in the United States and Europe [2]. In China, 654 formal landfills and thousands of informal landfills has been built by 2017. More than 55% of domestic waste in China is still disposed of by landfill [3,4]. Groundwater near landfills is easily contaminated. Leachate, which is produced by leaching and degradation of municipal solid waste deposited in landfills and contains a variety of pollutants in high concentration [5,6,7], is an important potential groundwater pollution source [8,9]. Heavy metals [10,11] and organic pollutants [12,13] in leachate can migrate for long distances in groundwater and accumulate in every link of the food chain, resulting in decreased cell activity, disturbance of human and animal endocrine systems, and even a variety of adverse health effects [14,15,16,17,18,19,20]. To eliminate or avoid the substantial risk to local groundwater resource user and the natural environment, and achieve effective management of drinking water source safety, it is essential to identify groundwater pollution around landfills and assess the corresponding health risks.

Hydro-geochemistry explains the formation of groundwater based quantitative comparison relationships as well as analysis of its element content, ionic balance and changes in hydro-chemical composition during water–rock interactions [21,22]. This method is often combined with multivariate statistics to identify groundwater pollution characteristics based on hydrogeologic investigations [23,24,25]. Shi et al. [26] analyzed the chemical types and hydrogeochemical processes of groundwater around Likeng landfill in Guangzhou using a Piper trilinear diagram and expanded Durov diagram, and identified the pollution sources of groundwater around the landfill by principal component analysis. Daniel et al. [27] analyzed physicochemical parameters in groundwater and identified the influence of landfill age, operation status and season on groundwater pollution. Makhadi et al. [28] applied hydrogeochemical methods to identify chemical variations in groundwater polluted by landfill sites, as well as the role of aquifer geological conditions in the process of groundwater quality deterioration and pollutant attenuation.

The “four step method” prescribed by the National Academy of Sciences and the United States Environmental Protection Agency (USEPA) is widely used in health risk assessments of various pollutants in groundwater [29]. Indeed, many environmental health risk assessments of groundwater pollution have been conducted using the four step method. Rakhi et al. [30] and Ma et al. [31] calculated the health risks of heavy metal pollutants and organic pollutants in the groundwater downstream of a landfill [32,33,34]. Controlled by the transportation of pollutants in groundwater, the health risk of leachate polluting groundwater around the landfill is continuously distributed. However, the results of health risk assessments are usually expressed as discrete risk values, which is not convenient for groundwater pollution risk control in areas downstream of landfills.

Red-beds are composed of purplish red or dark red sandstones and mudstones. Shallow weathering fissure zones developed in red-beds have low permeability [35]. The amount of shallow groundwater in red-bed areas is limited by the low permeability of shallow weathering fissure zones, and the total pumping volume of a single well cannot meet the basic needs of a large population. Therefore, protection of groundwater quality in red-bed areas is often ignored. However, the accumulated water supply scale of groundwater in red-bed areas is huge, and their regional water supply functions cannot be replaced. Most landfills in red-bed areas are located in suburbs and rural areas. Scattered residents are often distributed around landfills; however, these residents often have no choice but to extract groundwater from wells for drinking.

In view of this, there is an urgent need for relevant research on groundwater pollution identification and risk assessment around landfills in red-bed areas. In this study, a typical landfill in a red-bed area was taken as the research object. A hydrogeologic investigation and groundwater detection were then carried out in turn, after which the groundwater pollution characteristics of the landfill were identified using a hydrogeochemical method. The continuous spatial distribution of health risk caused by groundwater pollution in the landfill was then further characterized by a numerical simulation method combined with the four step method.

## 2. Study Area

### 2.1. Location and Meteorology

The study area is located in the red-bed hilly area in the middle of Sichuan Basin (104°26.7′–105°3.5′ E, 29°51.1′–30°18.5′ N), which is characterized by a tropical humid monsoon season and a mild climate. The average annual temperature is 17.4 °C and the average annual sunshine is 1284 h. The hottest month is August, when the average temperature is 26.5 °C, while the coldest month is January, when the average temperature is 6.5 °C. Based on observation of the long-term rainfall, the annual average rainfall is 961.3 mm, the maximum rainfall is 1290.6 mm, and the minimum rainfall is 725.2 mm. The rainfall varies greatly with seasons, with 72% occurring from June to September and only 6% from December to March.

The landfill investigated in this study is located in Ziyang city, Sichuan Province, China and has a total area of 127,000 m^2^. The middle part of the site is uplifted, while the south, west and east are surrounded by shallow hills that form two gullies that meet at the eastern edge of the site and extend northeastward. The site was used as a non-sanitary landfill from the 1980s to 2004 and then rebuilt as a sanitary landfill in 2005. The landfill applied 2.0 mm high-density polyethylene film as liner. In 2018, the landfill stopped filling and was temporarily covered with high-density polyethylene film. When it was closed, 3.25 million m^3^ of domestic waste had been deposited in the landfill.

A field investigation revealed that more than ten households are located about 260 m downstream of the northeast side of the landfill. Because there is no municipal water supply system, all residents currently drill wells to extract groundwater and drink directly without purification treatment. The water consumption of a single household is 2–5 m^3^/day, and the nearest distance between a well (PW1) and the landfill is only 190 m.

### 2.2. Hydrogeology

The clastic rock of the Suining Formation (J_2_sn) of Middle Jurassic, which contains shallow weathering fissure groundwater, is mainly exposed in the study area. Based on prior analysis of geologic, tectonic and hydrogeological conditions of the study area, the Suining Formation consists of sandstone and mudstone interbedding with different thicknesses. In the formation, the mudstone is purplish red and dark red, while the sandstone is grayish white and yellowish brown. The occurrence of rock strata is nearly horizontal, and the dip angle is about 5°. The strata may contain salt rock, calcite, gypsum and other minerals.

**Figure 1:**
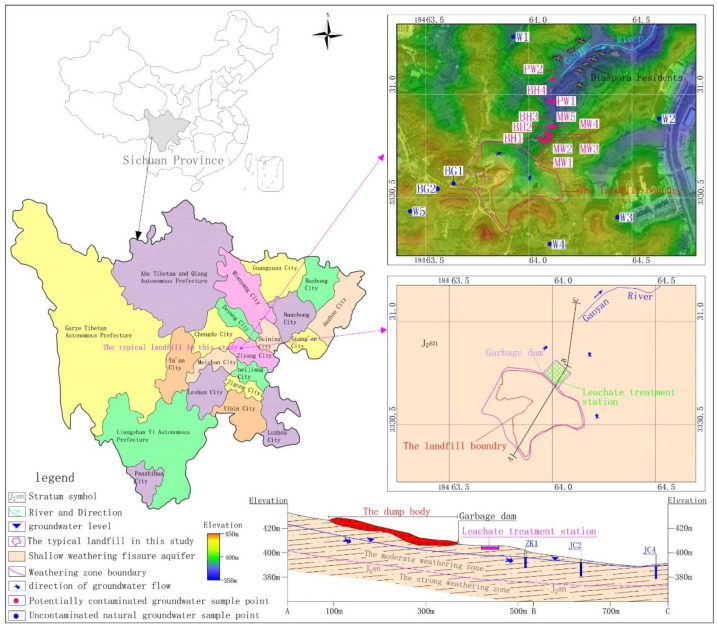
Location of the study area in Sichuan Province, China. The detailed inset in the upper right shows the topography of the study area, landfill layout and sampling points location. The detailed inset in the lower right shows the hydrogeological conditions of landfills.

Hydrogeological drilling and in-situ pressing water experiments around the landfill revealed that the thickness of the vadose zone at the top of the upstream hill on the southwest of the landfill ranges from 13.5 to 15.6 m (Figure 1). Additionally, the thickness of the vadose zone in the downstream gully on the northeast side ranges from 0.7 to 1.3 m. This zone is mainly composed of silty clay formed by weathering of the Suining Formation, as well as a small amount of plant roots and gravel particles. The depth of the strong weathering zone of the Suining Formation is 18.3–23.5 m, the horizontal permeability coefficient is 0.5–0.8 m/day, and the vertical permeability coefficient is 0.1–0.2 m/day. The strong weathering zone constitutes the phreatic aquifer of the study area. A moderate weathering zone underlies the strong weathering zone, and the vertical permeability coefficient is between 0.01–0.1 m/day. The moderate weathering zone mainly acts as a relatively impermeable layer. Rainfall infiltration is the only recharge source of phreatic aquifer. Controlled by the topography, the groundwater divide basically coincides with the surface water divide. After being recharged, the groundwater flows from high to low. The overall runoff direction of groundwater is from the divide and hillside to the northeast side of the landfill along the weathering fissure, and Gaoyan River receives the discharge of groundwater as a controlled drainage system. Because of the low permeability of the aquifer, groundwater leaching, runoff and discharge are generally slow.

## 3. Materials and Methods

### 3.1. Sampling and Laboratory Analysis

A total of twenty eight samples were collected, including nine unpolluted natural groundwater samples, seventeen groundwater samples potentially polluted by the landfill and two untreated raw leachate samples. Sampling points were set up around the landfill to detect groundwater and leachate (Figure 1). Sampling was conducted in January 2018, June 2020 and January 2021.

In 2018, seven unpolluted natural groundwater samples from drinking water wells W1-W5 and monitoring wells BG1 and BG2 and four samples potentially polluted by the landfill from boreholes BH1-BH4 were collected.

In 2020, an untreated raw leachate sample from the water inlet of the leachate treatment station, BG1 and six potentially polluted groundwater form monitoring wells MW1-MW4, and drinking water well PW1 and PW2 were collected. Except for an additional potentially polluted groundwater sample from monitoring well MW5, the other sampling sites in 2021 were the same as those in 2020. No rainfall events occurred in the three sampling processes.

Based on the characteristics of leachate and groundwater quality, 141 factors including inorganic salts, light metals, heavy metals, the comprehensive index, volatile organic compounds (VOCs) and semi VOCs (SVOCs) were selected as detection indicators. Before sampling, the wells or boreholes were continuously cleaned by pumps until constant values of electrical conductivity and pH was established. Fresh Baylor tubes were used in each well to avoid cross infection. Water table depth, pH and dissolved oxygen (DO) were determined directly during field sampling.

Samples for analyses of metals were packed in polyethylene bottles and acidified with nitric acid until the pH was between 1 and 2, to avoid hydrolysis and precipitation of metal ions and adsorption onto the container wall according to the water quality sampling–technical regulations for the preservation and handling of samples (HJ493–2009). Samples for the determination of inorganic, VOCs and SVOCs were packed in hard glass bottles, tightly capped and stored at 4 °C until analysis. Among the factors, Cl^−^, SO_4_^2−^, F^−^, NO_2_^−^ and NO_3_^−^ were analyzed by ion chromatography, while Na^+^, K^+^, Ca^2+^, Mg^2+^, Mn, Cd, Fe, Pb, As, Hg, Cu and Zn were analyzed by inductively coupled plasma mass spectrometry. Additionally, 57 VOCs and 58 SVOCs pollutants were screened and quantitatively analyzed by gas chromatography mass spectrometer based on NIST17. The IBM SPSS Statistics 22 and Microsoft Excel 2019 were used to compile the analysis results and to produce the descriptive statistics. Table 1 shows the monitoring methods and limits of each factor.

### 3.2. Simulation of Pollutants Spatial Distribution

The groundwater pollution transport simulation software Visual MODFLOW (Available online: https://www.waterloohydrogeologic.com/ (accessed on 15 April 2021)) was applied to build a hydrogeological numerical model to simulate the spatial distribution of pollutants in groundwater.

According to the hydrogeological drilling and investigation, the simulation area was generalized as a homogeneous heterogeneous stable groundwater flow system. The established groundwater numerical model set the surface divide around the landfill as the zero flux boundary and the Gaoyan River as the groundwater discharge boundary. The horizontal direction of the model was subdivided by a square grid with a side length of 10 m. The vertical direction was divided into two layers according to the permeability difference, a strong weathering zone and a medium weathering zone (from top to bottom). The hydrogeological parameters and solute transport parameters of the model were set according to hydrological geological tests and the results of previous studies (Table 2). After setting the parameters, the groundwater seepage field of the landfill was obtained by repeated calculations based on the water levels of drill holes and monitoring wells.

**Figure 2:**
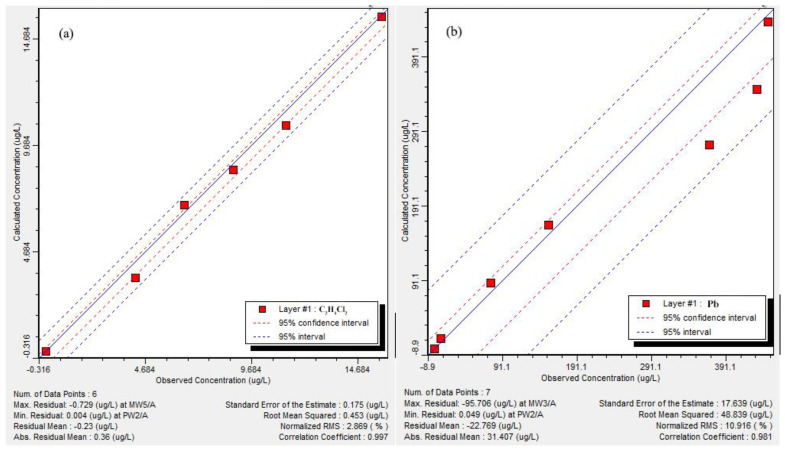
(**a**) Residual analysis of predicted and detected dichloroethane; (**b**) Residual analysis of predicted and detected Pb.

Based on the obtained seepage field, the infiltration flux of each pollution factor in the landfill was adjusted repeatedly by trial and error. The simulation results were compared with the detection results for pollutants in June 2021. Until the points of simulated versus observed values were around the X = Y line, there was a good fit between calculated and observed values (Figure 2a,b). It can be considered that the simulation results basically reflected the actual distribution of pollutants in the aquifer.

### 3.3. Risk Assessment

Based on the simulation results, the four step method was applied to evaluate the potential health risks associated with groundwater polluted by the landfill. This method consists of hazard identification, dose-effect assessment, exposure assessment and risk characterization. Based on the hazards or adverse health effects of exposure to certain chemicals, pollutants can be characterized as carcinogenic or non-carcinogenic. Carcinogenic and non-carcinogenic risks were characterized by a carcinogenic slope factor SF (kg·day/mg) and reference dose RfD (mg/(kg·day)) [36,37], respectively. This study both focused on the carcinogenic and non-carcinogenic risks posed by typical pollutants in groundwater downstream of the landfill.

Because groundwater was the only drinking water source of residents downstream of the landfill, the main exposure route of pollutants to humans was oral ingestion of groundwater. Therefore, the long-term daily exposure was calculated as follows:

(1) $${\mathrm{CDI}}_{\mathrm{oral}\text{-}\mathrm{water}}=\frac{C_{w}\times \mathrm{IR}\times \mathrm{EF}\times \mathrm{ED}}{\mathrm{BW}\times \mathrm{AT}}$$

where CDI_oral-water_ is the long-term daily exposure (L/(kg·day)); C_w_ is the concentration of a particular pollutant in groundwater (mg/(L·day)); IR is the daily water consumption (L/day); EF is the frequency in number of days exposed in a year (day/a); ED is the total years of exposure (a); BW is the weight of an adult (kg); and AT is the average exposure time (day).

Additionally, the non-carcinogenic risk (HI) of an individual pollutant in groundwater by oral ingestion was calculated as follows:

(2) $$\mathrm{HI}=\frac{{\mathrm{CDI}}_{\mathrm{oral}\text{-}\mathrm{water}}}{\mathrm{RfD}}$$

where RfD is the reference dose (mg/(kg·day)).

According to the Integrated Risk Information System of USEPA (2012), if the final value of HI < 1, then there is no impact on human health, but if HI > 1, there is a non-carcinogenic impact on human health.

The carcinogenic risk (R_o_) of a single pollutant in groundwater by oral ingestion was calculated as follows:

(3) $$R_{O}={\mathrm{CDI}}_{\mathrm{oral}\text{-}\mathrm{water}}\times \mathrm{SF}$$

where SF is carcinogenic slope factor (kg·day/mg).

Without considering the synergistic and antagonistic effects of various pollutants, the total carcinogenic risk (TR_o_) of each pollutant was the sum of the carcinogenic risk (R_o_) of each individual pollutant.

(4) $${\mathrm{TR}}_{o}=\sum_{1}^{n}R_{o}$$

The USEPA-recommended value is usually used to judge the risk of carcinogenesis. When TR_o_ is less than 10^−6^, the risk of cancer is considered to be relatively low, while a TR_o_ value of more than 10^−6^ indicates exposure is likely to result in cancer. Table 3 shows relevant parameters of health risk assessment.

## 4. Results

The collected uncontaminated groundwater samples were aerobic and alkalescent. The pH of the samples ranged from 7.1 to 7.6 and the total dissolved solids (TDS) ranged from 428 to 757 mg/L. TOC (Total Organic Carbon) ranged from 0.6 to 1.2 mg/L. The major cations in groundwater were Ca^2+^ and Mg^2+^, and the major anions were SO_4_^2−^ and HCO_3_^−^. Permanganate index (COD_Mn_), NH_4_^+^, NO_2_^−^, NO_3_^−^ and F^−^ were also detected, but at levels below the Standard for Ground Water Quality of China (GB/T14848–2017).

Downstream of the landfill, the groundwater was obviously influenced by the input of external chemicals, resulting in a change in the groundwater chemical environment. Anaerobic, aerobic, acidic and alkaline conditions all existed in the groundwater. The major cations in groundwater were converted into Na^+^ and Ca^2+^ and the major anions were converted into Cl^−^ and HCO_3_^−^. The maximum determined concentrations of COD_Mn_ and TOC were 14.5 mg/L and 7.2 mg/L, respectively. The concentrations of NH_4_^+^ and F in groundwater were basically consistent with those in natural groundwater. High levels of nitrate were only observed in a few sampling points, and the concentration of nitrite was very low. The levels of Mn, Pb and Fe were relatively high. Additionally, concentrations of As, Cu, Zn, Cd and Hg were higher than the background value in some sample points, but still far lower than the quality standard for groundwater of China and occasionally lower than the detection limit. With the exception of dichloroethane, chlorobenzene and chloroform, all organic pollutants investigated in this study were below the detection limits. Additionally, the dichloroethane, chlorobenzene and chloroform concentrations were low. Among these compounds, the detection rate of dichloroethane was highest, and its maximum concentration in groundwater was 15.8 µg/L. Table 4 shows the descriptive statistics of groundwater analysis.

## 5. Discussion

### 5.1. Hydrogeochemical Characteristics

**Figure 3:**
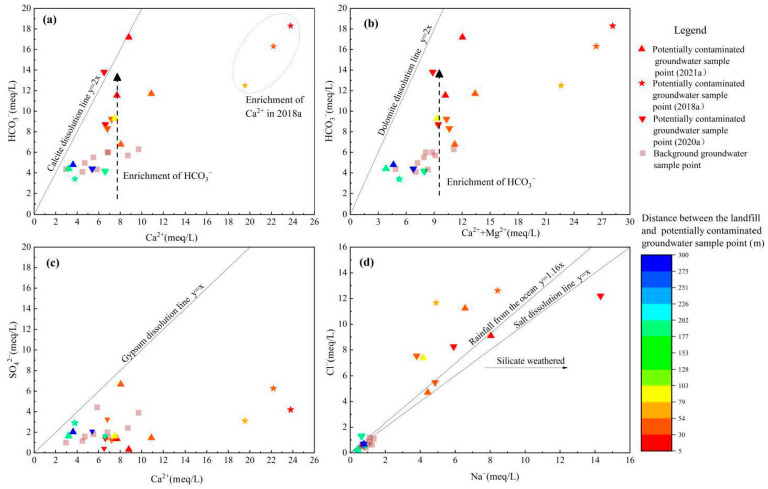
(**a**) γ (HCO_3_^−^)/γ (Ca^2+^) in the groundwater of the study area; (**b**) γ (HCO_3_^−^)/γ (Ca^2+^+Mg^2+^) in the groundwater of the study area; (**c**) γ (SO_4_^2^^−^)/γ (Ca^2+^) in the groundwater of the study area; (**d**) γ (Cl^−^)/γ (Na^+^) in the groundwater of the study area.

The ratio γ of the milliequivalent per liter of major ions is often used to analyze the source of the main chemical compositions in the groundwater. As shown in Figure 3c, γ (SO_4_^2−^)/γ (Ca^2+^) of natural groundwater sample points were located below gypsum dissolution line y = x. This indicated that SO_4_^2−^ originated from the dissolution of gypsum, while Ca^2+^ had other sources besides gypsum dissolution. Meanwhile, the contribution of gypsum to the level of Ca^2+^ in groundwater was limited. As shown in Figure 3a,b, γ (HCO_3_^−^)/γ (Ca^2+^) and γ (HCO_3_^−^)/γ (Ca^2+^ + Mg^2+^) of natural groundwater sample points were below and far away from calcite dissolution line y = 2x and dolomite dissolution line y = 2x. This further indicated that both calcite and dolomite dissolution existed in the shallow aquifer. Mg^2+^ originated from the dissolution dolomite. HCO_3_^−^ and Ca^2+^ mainly originated from the dissolution dolomite and calcite [38,39]. The low concentration of Na^+^ and Cl^−^ in natural groundwater indicated that Na^+^ and Cl^−^ were less likely to come from rock salt dissolution [40] (Figure 3d). The ratio of the milliequivalent per liter of Cl^−^ to Na^+^ ranged from 0.45 to 0.85, which further indicated that these ions originated from rainfall and a small amount of aluminosilicate dissolution [35,41].

**Figure 4:**
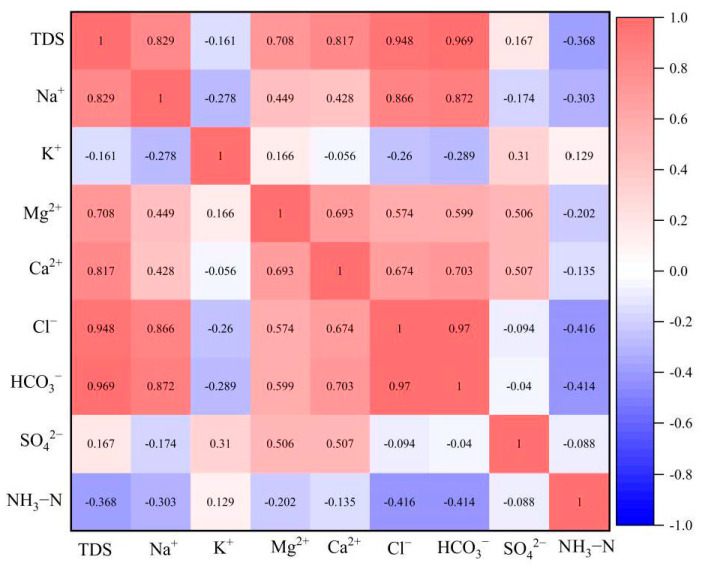
Pearson correlation analysis of major inorganic ions downstream of the landfill.

Pearson correlation analysis of the major ions in the groundwater downstream of the landfill showed that there was a strong correlation among TDS, Na^+^, Cl^−^ and HCO_3_^−^ in the groundwater downstream of the landfill, indicating that these compounds originated from the same source (Figure 4). The high concentrations of TDS, Na^+^, Cl^−^ and HCO_3_^−^ in the groundwater were consistent with the water quality of leachate (Table 4), indicating that the increased levels of TDS, Na^+^, Cl^−^ and HCO_3_^−^ in the groundwater downstream of the landfill were from leachate infiltration. Although the concentration of K^+^ in leachate reached 1370.5 to 1740.2 mg/L, the concentration of K^+^ in the groundwater was still very low and not significantly correlated with Na^+^, Cl^−^ or HCO_3_^−^. NH_4_^+^ had similar characteristics to K^+^. The aquifer in the study area is composed of strongly weathered sandstone and mudstone, and the content of illite, chlorite, montmorillonite and other clay minerals reaches 31.5% of the total minerals [42]. Clay minerals easily attenuate pollutants during migration with groundwater via ion-exchange adsorption. The ion selectivity occurs in the order Li < Na < Mg < Ca < Ba < NH_4_^+^ < K < Cs [43]. Therefore, the extremely low concentration of K^+^ and NH_4_^+^ in the groundwater downstream of the landfill can be attributed to the ion exchange adsorption of the aquifer.

It should be noted that the concentrations of Ca^2+^ and Mg^2+^ in the leachate from 2020 to 2021 were in the range of 24 to 61 mg/L and 36 to 105 mg/L, respectively, indicating that HCO_3_^−^ in the leachate mainly originated from the significant amounts of dissolved CO_2_ produced by the biodegradation of organic waste, rather than carbonate dissolution [24]. During this period, only HCO_3_^−^ was enriched in the groundwater downstream of the landfill, while Ca^2+^ did not increase significantly (Figure 3a,b). This phenomenon was consistent with the characteristics of leachate quality. In 2018, when the landfill was first shut down, Ca^2+^ in the groundwater downstream of the landfill increased significantly. When combined with the results of investigations of stabilization processes in anaerobic landfills, it is speculated that the reason for the increase of Ca^2+^ in the downstream groundwater was that waste newly deposited in 2018 was still in the transition to the acidification stage, and the acid leachate formed in this stage increased the dissolution of carbonate rock in the leachate, resulting in a simultaneous increase of Ca^2+^ and HCO_3_^−^ in the groundwater [44,45,46].

**Figure 5:**
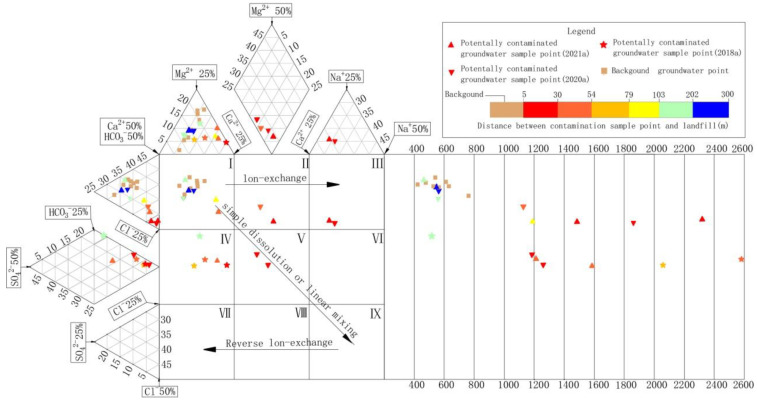
Expanded Durov diagram of groundwater in the study area.

An expanded Durov diagram was applied to further investigate the grouping of major ions and hydrogeochemical changes. There are nine regions in the expanded Durov diagram. Among these, Zone I, Zone V and Zone IX indicate the simple dissolution or linear mixing processes in groundwater, while Zone I, Zone II and Zone III indicate the ion exchange process and Zone IX, Zone VIII and Zone VII indicate the reverse ion exchange process [47,48,49]. As shown in Figure 5, the natural uncontaminated groundwater sampling points in the study area were distributed in zone I, and the chemical types of groundwater were HCO_3_-SO_4_-Ca-Mg, HCO_3_-Ca, and HCO_3_-SO_4_-Ca. The groundwater sampling points within 80 m downstream of the landfill were distributed in Zone V, Zone II and Zone III, and TDS increased to more than 1000 mg/L. Additionally, the chemical type of groundwater gradually evolved into Na-Ca-Cl-HCO_3_. These results showed that the main factors controlling the evolution of major ions in groundwater were the linear mixing and ion exchange of pollutants. As the distance from the landfill increased, the concentrations of TDS, Na^+^, Cl^−^ and HCO_3_^−^ decreased gradually. The TDS in the groundwater beyond 80 m downstream of the landfill decreased to 459–560 mg/L and returned to zone I in the expanded Durov diagram.

**Figure 6:**
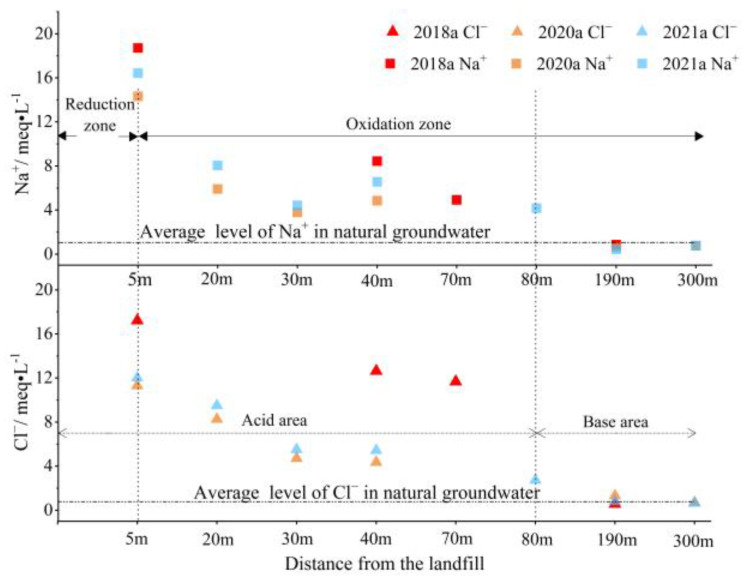
Milliequivalent concentration of Na^+^ and Cl^−^ in groundwater downstream of the landfill.

**Figure 7:**
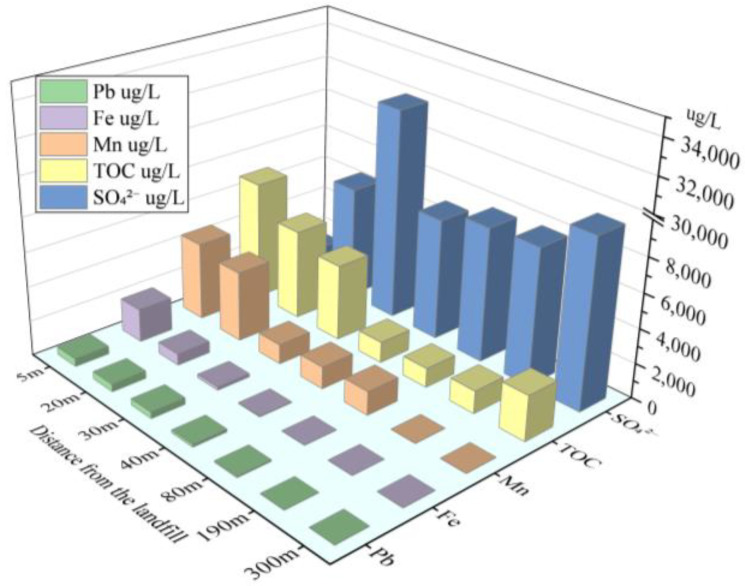
Concentration distribution of pollutants downstream of the landfill in 2021a.

Because there was no salt rock in the natural aquifer, Na^+^ and Cl^−^ can be used as rapid identification factors of landfill groundwater pollution in the study area. Because of pollution from the landfill, the ratios of the milliequivalent per liter of Na^+^ and Cl^−^ in the groundwater downstream of the landfill to those in natural groundwater reached 17.7 and 22.6, respectively. As the distance from the landfill increased, the milliequivalent per liter of Na^+^ and Cl^−^ decreased. The concentrations of Na^+^ and Cl^−^ in the groundwater 190 m downstream of the landfill were basically the same as those in the natural groundwater (Figure 6), indicating that groundwater was weakly affected by the landfill. The extremely low concentration of heavy metals and organic pollutants further confirmed this finding (Figure 7). Therefore, the plume boundary of leachate pollution was 190 m downstream of the landfill.

### 5.2. Groundwater Pollution Characteristics

Leachate with weak alkalinity and strong reduction leaking into a natural aquifer will change the oxidation-reduction and acid-base environment of the groundwater downstream of the landfill, thus affecting the migration and degradation characteristics of pollutants [43,50,51]. The DO in the groundwater 5 m downstream of the landfill was 0.78 mg/L, which indicated that the water was anaerobic and the main chemical processes in the groundwater were based on anaerobic degradation. The area from the landfill to 5 m downstream of the landfill was defined as the reduction zone. The higher concentration of TOC detected in the reduction zone further confirmed that electron donors were consuming dissolved oxygen in groundwater in the reduction zone (Figure 7). Beyond 5 m, the DO increased from 1.53 to 4.89 mg/L with increasing distance from the landfill, similar to previous findings for unconfined aquifers [52], indicating that the main chemical process in the groundwater in this area was aerobic oxidation; therefore, this region was defined as the oxidation zone.

The groundwater within 80 m downstream of the landfill was weakly acidic (pH ranged from 6.51 to 6.83), while that beyond 80 m downstream of the landfill was weakly alkaline, which was consistent with the natural groundwater in the region. The raw leachate of the landfill was weakly alkaline, with pH values ranging from 8.23 to 8.35, indicating that the acid-base characteristics of leachate are not the only factor controlling the acid-base characteristics of groundwater downstream of the landfill. The TOC of groundwater in the acidic area ranged from 4.4 to 7.2 mg/L, while that in the alkaline area ranged from 1.1 to 2.6 mg/L. When combined with the dissolved oxygen concentrations observed in the reduction and oxidation zones, it is speculated that the main reason for the transformation of groundwater into an acidic environment downstream of the landfill is that a high amount of organic matter decomposed under anaerobic conditions and produced a large number of small molecular organic acids [53].

Among various groundwater pollution factors, the migration and attenuation of sulfate, Fe, Mn and Pb in groundwater were affected by the redox conditions of groundwater and changes in the acid-base environment. In the groundwater downstream of the landfill, sulfate first increased, then decreased as the distance from the landfill increased (Figure 7). This may have occurred because the sulfide produced by anaerobic reaction of leachate and organic compounds in the reduction zone is gradually oxidized to sulfate as groundwater migrates downstream, resulting in the accumulation of sulfate in the downstream groundwater [54]. With the migration of iron from the reduction zone to the oxidation zone, the concentration of iron decreased rapidly from 1920.1 to 5.5 µg/L. The blocking effect of the aquifer on iron can be attributed to precipitation, ion exchange adsorption and dilution [52]. Additionally, the negative correlation between iron and dissolved oxygen indicated that ferrous iron was oxidized to form ferric hydroxide, which may also explain the rapid decrease of iron concentration in the aquifer [43,54]. Mn in the groundwater near the landfill experienced a similar attenuation process as iron. However, iron oxidizes and precipitates before Mn [55,56], therefore, the attenuation of Mn was smaller than that of iron. The concentration of Mn in groundwater increased slightly from 30 to 80 m downstream of the landfill. This may indicate that the organic matter in the pollution plume oxidized the Mn deposits in the aquifer, thereby increasing the concentration of Mn in the pollution plume [57]. The concentration of Pb ranged from 334.4 to 525.3 µg/L within 40 m downstream of the landfill, far exceeding the Standard for Ground Water Quality of China. Pb in landfill leachate, which originates from waste batteries, fluorescent lamps and Pb pipes, may cause serious harm to human health. Indeed, even very low concentrations of Pb can affect the human brain and nervous system for a long time [58]. The level of Pb decreased as the distance from the landfill increased and the area of Pb enrichment in groundwater was consistent with that of acidic groundwater (Figure 6 and Figure 7). These findings indicated that the acidic groundwater environment promoted the release of adsorbed Pb into groundwater, thus aggravating heavy metal pollution downstream of the landfill.

### 5.3. Groundwater Health Risk Assessment

The above analysis shows that the landfill has caused heavy metal, inorganic and organic pollution of the groundwater downstream of the landfill, and that the main pollution factors are HCO_3_^−^, Na^+^, Cl^−^, Mn, Pb, dichloroethane, chlorobenzene and chloroform. The relevant dose-effect assessment studies show that Mn and Pb mainly cause non-carcinogenic harm to the human body. Although the levels of dichloroethane, chlorobenzene and chloroform detected were very low, the synergistic effects of various pollutants and their accumulation in organisms could have adverse effects and may even cause cancer. Therefore, Mn, Pb, dichloroethane, chlorobenzene and chloroform were selected for risk assessment. The carcinogenic slopes and reference dose values of each factor are shown in Table 5 [59,60].

**Figure 8:**
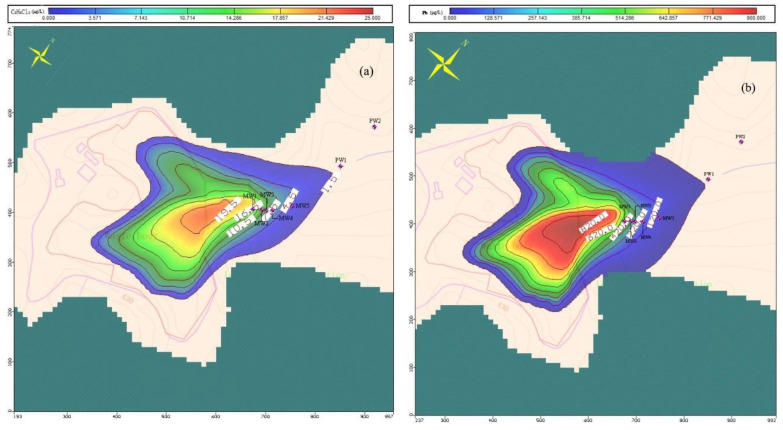
(**a**) Simulation result of spatial distribution of dichloroethane; (**b**) Simulation result of spatial distribution of Pb.

According to the simulation results (Figure 8a,b), the migration distances of Pb and dichloroethane were largest (152 m and 176 m, respectively) among the selected factors, followed by Mn, which had a migration distance of 131 m. Chlorobenzene and chloroform were mainly distributed 35 m downstream of the landfill, and the pollution plume area was about 3000 m^2^.

**Figure 9:**
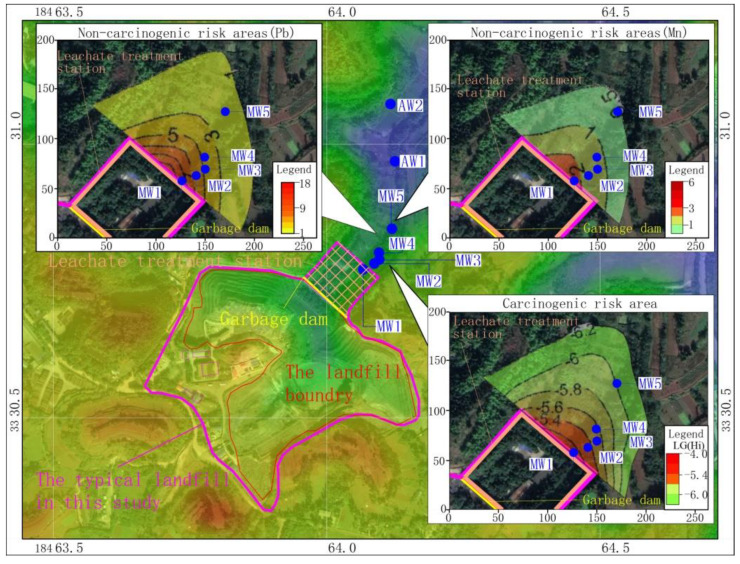
Spatial distribution of health risk assessment results.

Based on the simulation results, the non-carcinogenic risks caused by Mn, Pb, dichloroethane, chlorobenzene and chloroform and the total carcinogenic risks caused by dichloroethane, chlorobenzene and chloroform were calculated (Figure 9).

The maximum non-carcinogenic risk of Mn in the groundwater downstream of the landfill was 2.9 and the area with a HI value of more than 1 extended from the landfill to 50 m downstream, giving a total area of 3565 m^2^. The maximum non carcinogenic risk of Pb in the groundwater downstream of the landfill was 11.5 and the area with an HI value of more than 1 extended to 130 m downstream of the landfill, covering an area of 15,485 m^2^. The boundary of the area with a HI value of more than 1 was only 60 m away from a well that was used as the drinking water source for residents. Dichloroethane, chlorobenzene and chloroform are not carcinogenic in humans; however, the total cancer risk TR_o_ was as high as 7.9 × 10^−6^. The area with a TR_o_ value of more than 10^−6^ extended to 106 m downstream of the landfill, giving a total area of 11,414 m^2^. The boundary of the area with a TR_o_ of more than 10^−6^ was only 84 m away from a drinking water well.

## 6. Conclusions

To ensure the water security of groundwater for residents in red-bed regions with low permeability, this study took a typical red-bed region landfill as an example for a groundwater pollution investigation and health risk assessment. The results showed that the hydrogeochemical method can effectively identify the characteristics of pollutants migration from a landfill. The natural groundwater in the study area contained dissolved calcite, dolomite and a small amount of gypsum, which resulted in weakly alkaline groundwater with hydro-chemical types of HCO_3_-SO_4_-Ca-Mg and HCO_3_-SO_4_-Ca. After being polluted by the landfill, the TDS levels in the groundwater increased and hydro-chemical type evolved to be Na-Ca-Cl-HCO_3_. Accordingly, Cl^−^ and Na^+^ can be used as rapid identification factors for groundwater pollution in the study area. The pollution plume of landfill extended from the landfill to 190 m downstream, and the most polluted area was within 80 m downstream of the landfill. The rapid identification, detection of organic pollution factors and numerical simulation results can be mutually supportive. Additionally, the health risk assessment showed that the groundwater pollution caused by the infiltration of leachate from the landfill has indeed caused health risk. The author has proposed to the relevant decision-making departments that the drinking water sources of residents near the landfill should be replaced. Additionally, source–process–end control measures should be implemented to ensure the future safety of groundwater drinking sources distributed further downstream of the landfill. In view of the limited migration distance of pollutants in the red-bed area, the landfill can be closed in situ by using a coverage system to minimize the generation and infiltration of leachate [61,62]. The aquifer will act as a natural treatment plant to naturally attenuate pollutants. However, it will also be necessary to construct a network for long-term monitoring of groundwater quality.

## Figures and Tables

**Table 1:** Analytical methods and minimum detection values.

No.	Monitoring Factors	Groundwater		
		Detection Method	Instrument	Minimum Detectable Value
1	pH	Portable PH meter	-	-
2	TDS	Weighing method	-	5 mg/L
3	COD_Mn_	Alkaline potassium permanganate oxidation method	-	0.5 mg/L
4	Total hard	EDTA tltrimetric method	-	5 mg/L
5	NH_4_^+^	Salicylic Acid Spectrophotometry	UV-Vis-TU-1901	0.01 mg/L
6	TOC	Non-dispersive infrared absorption method	MHY-26359	0.1 mg/L
7	DO	Point-of-care Testing	BDO-200A Dissolved Oxygen Meter	-
8	Cl^−^	Ion chromatography	IC-1.925.0020	0.007 mg/L
9	SO_4_^2−^			0.018 mg/L
10	F^−^			0.006 mg/L
11	NO_3_^−^			0.016 mg/L
12	NO_2_^−^			0.016 mg/L
13	HCO_3_^−^	Acid-base indicator titration	-	0.19 mg/L
14	CO_3_^2−^			0.19 mg/L
15	Na^+^	Inductively coupled plasma-mass spectrometry	ICP-MS-7800	6.36 μg/L
16	K^+^			4.5 μg/L
17	Ca^2+^			6.61 μg/L
18	Mg^2+^			1.94 μg/L
19	Mn			0.12 μg/L
20	Cd			0.05 μg/L
21	Fe			0.82 μg/L
22	Pb			0.09 μg/L
23	As			0.12 μg/L
24	Hg			0.04 μg/L
25	Cu			0.08 μg/L
26	Zn			0.67 μg/L
27	VOCs	Purge and Trap-GC-MS	P&T-GC/MS-8860/5977B	VOCs include 57 factors. The minimum detectable value of epichlorohydrin is 5 μg/L. The minimum detectable values of other pollutants are between 0.2 and 2.2 μg/L.
28	SVOCs	GC-MS	GC/MS-7890B/5977B	SVOCs include 58 factors. The minimum detectable values of 3,3-dichlorobenzidine,4-chloroaniline, dimethoate and 2-methyl-4,6-dinitrophenol are 16 μg/L, 20 μg/L, 20 μg/L and 24 μg/L respectively. The minimum detectable values of parathion-methyl, 2,4-dinitrophenol, 2-nitroaniline, 3-nitroaniline and malathion are between 40 and 50 μg/L. The minimum detectable values of other pollutants are between 0.2 and 10 μg/L.

**Table 2:** Model parameter values used in numerical simulations.

Layering	Average Annual Rainfall	Rainfall Infiltration Coefficient	Total Porosity	Specific Yield	Hydraulic Conductivity		Longitudinal Dispersion	Transverse Dispersion
					Kx,Ky	Kz		
First layer	961.3 mm	0.12	0.15	0.1	0.65 m/day	0.14 m/day	1.54 m	0.15 m
Second layer	-	-	0.08	-	0.08 m/day	0.03 m/day	-	

**Table 3:** Relevant parameters of health risk assessment.

Symbol	Name	Unit	Recommended Values (Adult)	Symbol	Name	Unit	Recommended Values (Adult)
Cw	Concentration of i in groundwater	mg/(L/day)	Measured value	ED	Total years of exposure	a	40~70
IR	Daily water consumption	L/day	3.53	BW	weight	kg	67.3
EF	Exposure frequency number of days exposed in a year	day/a	365	AT	Average exposure time	d	10,950–25,550

**Table 4:** Descriptive statistics of groundwater analysis.

Number	Monitoring Factors	Unit		Uncontaminated Natural Groundwater						Potentially Contaminated Groundwater					Leachate		Standard Value
			Number of Samples	Average.	Min	Max	Median	Over Standard Rate	Number of Samples	Average.	Min	Max	Median	Over Standard Rate	Min	Max	
1	pH	-	9	7.3	7.1	7.6	7.3	0.0%	17	7.00	6.51	7.91	6.83	0.0%	8.23	8.35	6.5–8.5
2	Total hard	mg/L	9	405	336	555	384	23.5%	17	592	191	1412	513	70.6%	198	579	450
3	TDS	mg/L	9	560	428	757	544	0.0%	17	1385	459	2947	1248	70.6%	15,635	17,095	1000
4	Na^+^	mg/L	9	22.2	14.9	30.9	22.9	0.0%	17	141	10	430	112	17.6%	1980	1990	200
5	K^+^	mg/L	9	2.1	1.0	5.5	1.8	-	17	2.0	0.6	4.3	1.7	-	1370.5	1740.2	-
6	Ca^2+^	mg/L	9	124.4	59.2	194.0	123.0	-	17	189	76	476	145	-	24	61	-
7	Mg^2+^	mg/L	9	21.8	4.9	31.0	23.1	-	17	31	8	53	30	-	36	105	-
8	Cl^−^	mg/L	9	25.5	13.9	42.1	21.7	0.0%	17	228	20	611	192	41.2%	2561	3030	250
9	SO_4_^2−^	mg/L	9	92.2	48.2	187.0	81.2	0.0%	17	117.5	16.1	320.0	79.3	11.7%	50.2	171.8	250
10	HCO_3_^−^	mg/L	9	327.4	250.0	384.0	342.0	-	17	591	207	1116	563	-	3560	4230	-
11	NH_4_^+^	mg/L	9	0.0	0.0	0.2	0.0	0.0%	17	0.1	0	0.3	0.1	0.0%	872.4	1090.1	0.5
12	NO_2_^−^	mg/L	9	0.0	0.0	0.10	0.0	0.0%	17	0.006	0.000	0.036	0.000	0.0%	0.000	0.345	1
13	NO_3_^−^	mg/L	9	9.1	5.5	15.4	8.3	0.0%	17	8.649	0.070	66.800	0.880	11.7%	4.532	7.819	20
14	COD_Mn_	mg/L	9	0.7	0.0	1.1	0.8	0.0%	17	5.1	0.0	14.5	4.6	58.8%	1163.3	1180.5	3
15	F	mg/L	9	0.5	0.4	0.6	0.4	0.0%	17	0.318	0.240	0.650	0.280	0.0%	1.6	3.5	1
16	DO	mg/L	2	5.11	5.01	5.23	-	-	13	3.02	0.78	4.89	3.01	-	0.00	0.00	-
17	TOC	mg/L	2	0.90	0.6	1.2	-	-	13	3.3	1.1	7.2	2.6	-	1150.0	1453.8	-
18	Mn	µg/L	9	0	0	0	0	0.0%	17	1832.3	0.8	4835.4	1324.6	82.3%	1140.2	2900.5	100
19	Fe	µg/L	9	0	0	0	0	0.0%	17	477.5	2.8	2568.3	58.3	35.3%	2358.4	4752.7	300
20	Pb	µg/L	9	0	0	0	0	0.0%	17	222.1	0.0	525.3	153.2	64.7%	242.3	631.5	10
24	Dichloroethane	µg/L	2	0	0	0	-	0.0%	13	5.5	0.0	15.8	4.2	0%	45.7	46.2	20
25	Chlorobenzene	µg/L	2	0	0	0	-	0.0%	13	1.5	0.0	7.4	0.0	0%	15.7	18.2	300
26	Chloroform	µg/L	2	0	0	0	-	0.0%	13	1.0	0.0	4.3	0.0	0%	10.9	11.7	60

**Table 5:** Carcinogenic slope factors and reference dose values of toxic and hazardous pollutants.

Pollutants	Unit	Mn	Pb	Chlorobenzene	Dichloroethane	Chloroform
SF	kg·d/mg	1.4 × 10^−1^	3.5 × 10^−3^	2.0 × 10^−2^	2.0 × 10^−1^	1.0 × 10^−2^
RfD	mg/(kg·d)	-	-	-	5.7 × 10^−3^	3.1 × 10^−2^
